# Strangulated Hernia

**Published:** 1931

**Authors:** Noël Kemm

**Affiliations:** Honorary Surgical Registrar, Bristol Royal Infirmary


### STRANGULATED HERNIA.

BY

NOËL KEMM, M.B., B.S., F.R.C.S.,

*Honorary Surgical Registrar, Bristol Royal Infirmary.*

---

THE following data have been drawn from an analysis of the cases of strangulated hernia* (inguinal, femoral and umbilical) admitted to the Bristol Royal Infirmary during the years 1927-1929 inclusive :—

*Age incidence.*—

| | *Inguinal.* | *Femoral.* | *Umbilical.* | |
|---|---|---|---|---|
| First decade | 4 | 0 | 0 | = 4 |
| Second ,, | 0 | 0 | 0 | = 0 |
| Third ,, | 1 | 0 | 0 | = 1 |
| Fourth ,, | 2 | 1 | 0 | = 3 |
| Fifth ,, | 5 | 7 | 0 | = 12 |
| Sixth ,, | 10 | 5 | 3 | = 18 |
| Seventh ,, | 15 | 7 | 2 | = 24 |
| Eighth ,, | 5 | 9 | 2 | = 16 |
| Ninth ,, | 2 | 1 | 0 | = 3 |
| Totals | 44 | 30 | 7 | = 81 |

*Sex incidence.*—

| | *Inguinal.* | *Femoral.* | *Umbilical.* |
|---|---|---|---|
| Male | 39 | 9 | 2 |
| Female | 5 | 21 | 5 |

*Duration of hernia history.*—Of the cases (58) in which a history was available on this point, in nine cases the hernia had been noticed for less than a

* Made at the request of the Association of Surgeons of Great Britain and Ireland.

week, in five for a period of between one week and one year, and in forty-four cases of over one year.

In one case of femoral hernia in a woman aged 51 years at the time of strangulation a radical cure had been performed six years previously; she stated that the hernia recurred almost at once. In one case of inguinal hernia the patient said that he had been operated on while in the army; his hernia recurred in less than a week from the time of his operation.

*Principal symptoms.*—It is perhaps worthy of note that in three cases in this series the patient complained of retention of urine; in one case it appears to have been the sole symptom of which he complained. This case is referred to again later on in this report. In the other two cases additional symptoms were complained of, which could be definitely referred to the irreducible hernia that was present. In this connection I suggest that in cases of retention of urine which are associated with an irreducible hernia it would be sound practice to explore the hernial sac.

*Anæsthetics.*—These were as follows:—

| | | |
|---|---|---|
| C. and E. .. .. | 73 | cases. |
| Ether .. .. .. | 1 | ,, |
| Gas and oxygen .. | 5 | ,, |
| Novocain .. .. | 2 | ,, |

In no case was a spinal anæsthetic given.

*Operation.*—Throughout this series of cases a modified Bassini operation was always done in cases of inguinal hernia, where the condition of the patient permitted it; and similarly in cases of femoral hernia a Lotheissen's operation was performed. When a resection was necessary and an anastomosis made, an end to end union was effected in every case except one, which was done by the side to side method.

*Mortality.*—Out of the 74 cases of inguinal or femoral hernia ten, or 13·5 per cent., died. In six of these cases the patient was profoundly ill on admission, and died within a few hours of the operation; it is difficult to imagine by what means their lives could have been saved. In two of these cases an incision was made into the bowel and fluid contents were allowed to escape; in one case it was apparently done on purpose, in the other case it is not clear whether it was done purposely or accidentally on opening the sac.

In the remaining four fatal cases it is possible to wonder whether some alteration in treatment might have achieved a different result. They were:—

(*a*) One case of "reduction *en masse*" of a strangulated hernia during the course of an operation.

(*b*) One case where apparently the patient's sole complaint on admission was retention of urine (the bladder being at the level of the umbilicus). It is interesting that it was found to be impossible to catheterize this man under an anæsthetic, and a supra-pubic incision had to be made; proving that there were good grounds for supposing that he might be suffering from retention of urine, without taking his irreducible hernia into account. An interval of some thirty-six hours elapsed between the supra-pubic incision and the exploration of the hernial sac, when the whole of the ascending colon was found to be gangrenous, and the patient died in a few hours after resection had been carried out.

(*c*, *d*) Two cases of manual reduction of strangulated hernia. In the first case forty-eight hours after the manual reduction of a strangulated hernia the patient developed peritonitis, and on laparotomy a loop of gangrenous intestine with three perforations was found. In the second the patient developed signs of an acute abdominal crisis subsequent to the manual reduction of a strangulated hernia. On exploration a gangrenous mass of intestine and mesentery was found to be bleeding profusely. These two cases suggest that house-surgeons and students should be taught on no account to attempt the manual reduction of a hernia exhibiting signs of strangulation.

Out of the seven cases of umbilical hernia two died, giving a mortality of 28·6 per cent.

*Post-operative complications, etc.*—In this series there was no case of perforation, intestinal hæmorrhage, or femoral thrombosis subsequent to the operation.

The only important pulmonary sequelæ were pneumonia in one case, that of a male aged 80, who died two days after the operation; and severe bronchitis in another patient, who was transferred to medical ward and recovered.

In one case 40 c.c. of anti-gas-gangrene serum were given; the patient recovered.

The average stay in hospital for inguinal and femoral cases was 13 days; in the umbilical cases 25 days.

